# Gender mainstreaming at 25 years: Toward an inclusive, collaborative, and structured research agenda

**DOI:** 10.7189/jogh.14.04011

**Published:** 2024-01-26

**Authors:** Kelsi Caywood, Gary L Darmstadt

**Affiliations:** 1 Department of Sociology, University of Michigan, Ann Arbor, Michigan, USA; 2 Department of Pediatrics, Stanford University School of Medicine, Stanford, California, USA

## Abstract

**Background:**

Gender mainstreaming has been central to the development agenda for advancing gender equality globally for nearly three decades. We examined key learning across gender mainstreaming models and experiences and assess key successes and challenges in actualising gender mainstreaming’s transformative potential, in order to inform future research agendas.

**Methods:**

We reviewed 27 years of peer-reviewed literature on gender mainstreaming (1995–2022) and described scholarly publishing trends on the topic based on a set of 528 articles and bibliographic data retrieved from the Scopus database and supplemental coding. The review provides a thematic synthesis of the extant literature, assessing the evidence base to identify gaps and opportunities for future research and collaboration with practitioners. We also contextualise recent research by tracing common threads of scholarly and practitioner discussions over the last two decades.

**Results:**

Publications on gender mainstreaming have increased, primarily from authors with European and USA academic affiliations and funding. Gender mainstreaming in the health and law and policy sectors has been researched most frequently. Trends in co-authorship suggest increasing collaboration among academics, yet limited collaboration among researchers and practitioners. Widespread low citation counts raise concerns about engagement with the literature. Key challenges in gender mainstreaming identified include conceptual clarity, academic-practitioner disjunctures, politics, leadership and organisational culture, men’s roles, intersectionality, monitoring and evaluation, and public health sectoral concerns.

**Conclusions:**

The gender mainstreaming literature has expanded considerably over the last 25 years, yet there remain critical knowledge gaps, theoretical inconsistencies, weak research methods and evaluation processes, and implementation challenges. Funders, researchers, and practitioners have failed to prioritise bridging north-south and academic-practitioner divides in gender mainstreaming policy, programmes, and research. Integration of intersectionality also remains nascent. A more inclusive, collaborative, and structured research agenda on gender mainstreaming is needed to effect greater change in the face of persistent and new challenges. Engaging and empowering regional women’s organisations, collaborative learning and research programmes, and joint research and advocacy groups; implementing gender-attuned editorial policies; and incorporating gender mainstreaming in educational curricula are recommended.

Over 25 years ago, the Beijing Declaration and Platform for Action called for ‘governments and other actors (to) promote an active and visible policy of mainstreaming a gender perspective in all policies and programmes so that, before decisions are taken, analysis is made of the effects on women and men, respectively’ [1]. The Platform solidified ‘gender mainstreaming’ as a priority in the global development agenda, building on decades of women’s advocacy and incipient national-level efforts.

Despite its political achievements, the Beijing Platform’s vision is regarded by the development community as largely unmet in practice: global and local international development actors took several missteps in institutionalisation, implementation, and impact. In the last decade, critics argued that the aspirations of gender mainstreaming achieving a ‘utopian vision of change’ transitioned into tempered optimism about its ‘slow revolution’ of ‘continuous and consistent work... to induce transformational change in favour of gender justice’ [2,3]. The challenges and successes of gender mainstreaming as a global policy are well-recorded in the academic literature. At the 25th anniversary of the Beijing Platform, it is crucial to understand how a revitalised research agenda on gender mainstreaming can effect greater change in the face of persistent and new anti-gender equality movements.

Here we explore how well the existing literature captures the learning across gender mainstreaming models and experiences. Has gender mainstreaming, broadly conceived, lived up to its transformative potential? We focus on gender analysis in development policies and programmes, and on the health sector as both a primary context in which gender mainstreaming is applied and an emphasis in the multisector literature. We examine the theories of change represented in the literature which have been adopted in diverse institutional contexts. Specifically, we aim to: identify gaps and opportunities for future research and action on gender equality; provide an account of changes and trends after 2015, when the last high-level overview [4] of gender mainstreaming was published, to our knowledge; and contextualise recent research by tracing common threads of the scholarly and practitioner discussions occurring over the past 25 years.

## METHODS

We conducted an initial search on the Scopus database of peer-reviewed literature for articles published from January 1995 through December 2022 with ‘gender’ and ‘mainstreaming’ both contained in the article title (not sensitive to order). The initial search retrieved 569 documents, excluding foreign-language articles and specific types of non-research manuscripts (such as errata). We extracted the articles and their bibliographical data, which then underwent a preliminary review of all titles and abstracts, providing a macro-level sense of the literature trends and ensuring they conformed with the substantive scope of the review. We excluded articles we deemed irrelevant, such as those where ‘gender’ and ‘mainstreaming’ were included in an article title, but the article did not discuss gender mainstreaming. We also merged duplicate articles appearing in multiple journals or twice within the database into a single entry. In total, 528 articles were included for analysis.

All 528 articles were part of the empirical analysis of publishing trends and abstract review, with a smaller subset of 50 articles selected for full review. We extracted information on general publication and thematic trends, including primary funding sources, countries, and academic institutions from which the works originate. We coded additional article information such as the research methods used, sector(s) addressed, and country of programme or policy implementation, if applicable. Articles were assigned as being produced within an institution according to the institutions listed for each author of an article. Country information on publication source was based on the institutional address(es) for the article’s author(s). To assess collaboration, we coded author institutions listed in each article according to World Bank income classifications. We then selected a smaller subset of 50 articles based on titles, abstracts, keywords, and their conceptual and methodological contributions to the body of literature on gender mainstreaming. We closely reviewed the full text of these articles to inform the thematic analysis focused on the successes and challenges of gender mainstreaming.

## RESULTS

### Publishing trends

**Figure 1:**
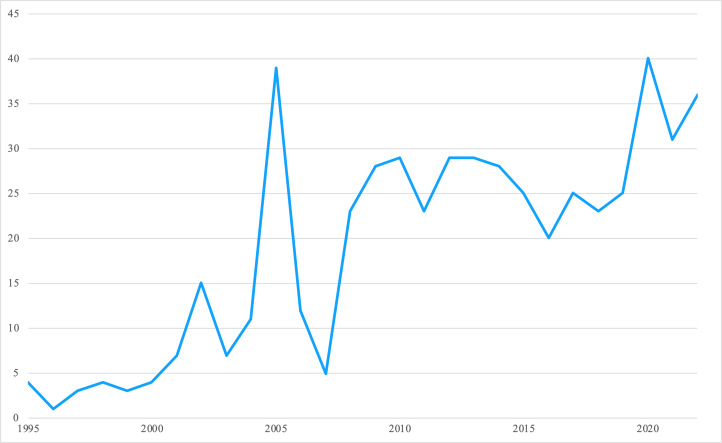
Gender mainstreaming articles published (number) by year.

The number of articles published annually on gender mainstreaming has increased nearly 10-fold since 1995, peaking at 40 articles in 2020 (**Figure 1**). Spikes in the literature reflect themed journal issues and major milestones such as 2005 and 2010, the five- and ten-year anniversaries of the Beijing Platform.

**Figure 2:**
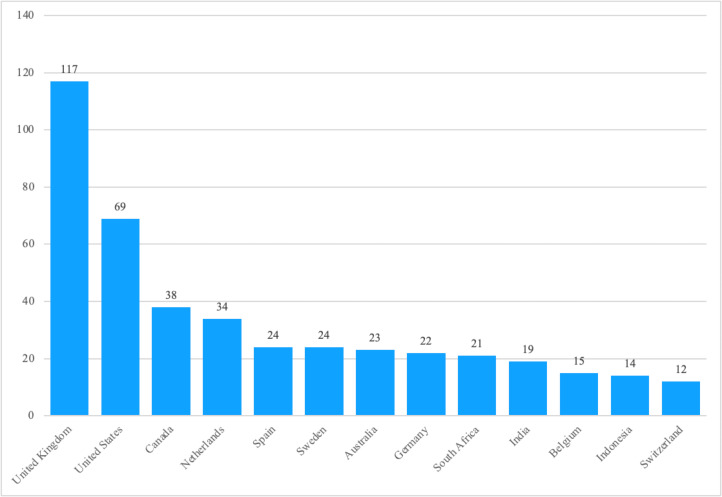
Number of gender mainstreaming publications by country of origin from 1995 through 2022, for countries with ten or more gender mainstreaming articles published.

Most authors contributing to gender mainstreaming literature held primary affiliations with academic institutions. About one-third had previous and/or concurrent practitioner experience, as determined by searches of authors’ backgrounds. Scholars based in the UK and the USA published most frequently (**Figure 2**). The largest reported funding sources for efforts that led to academic publishing were the UK Economic and Social Research Council, the European Commission, the Horizon 2020 Framework Program, the Social Sciences and Humanities Research Council of Canada, the Academy of Finland, the Consortium of International Agricultural Research Centers, the UK Department for International Development, and the World Bank Group. However, these funders and affiliations still represented a small overall percentage of the diverse literature on gender mainstreaming. Several other major development organisations and implementing organisations and foundations (e.g. the USA Agency for International Development, the International Monetary Fund, the Bill and Melinda Gates Foundation, Family Health International (FHI) 360) were infrequently involved in generating the published literature, with all but eight organisations supporting the development of two or fewer publications (Table S1 in the **Online Supplementary Document**); these institutions are more commonplace in the grey literature and practitioner publications. Authors’ university affiliations reflected these funding sources, with Europe as a primary research hub. In order of frequency, university affiliations included the Radboud University, the Simon Fraser University, Cardiff University, the University of the West of England, the University of Liverpool, the Complutense University of Madrid, the London School of Economics and Political Science, the University of Auckland, the University of Antwerp, and the Royal Tropical Institute (Table S2 in the **Online Supplementary Document**).

Articles often focussed on gender mainstreaming within a particular region, country, or organisational context. Overall, over 170 distinct actors or geographic areas were specified in the literature, demonstrating the diverse contexts in which gender mainstreaming has been applied and studied (Table S3 in the **Online Supplementary Document**). Among them, Europe is most represented in the literature; approximately one-fourth of the articles focussed on European countries or institutions.

The most represented sectors in articles on gender mainstreaming were governance, health, education, and peacekeeping and security (Table S4 in the **Online Supplementary Document**). The leading sectors reflect early adopters of gender mainstreaming, such as water, sanitation, and hygiene (WASH) and employment, as well as trending sectors like climate change and the environment and urban planning.

### Collaboration

**Figure 3:**
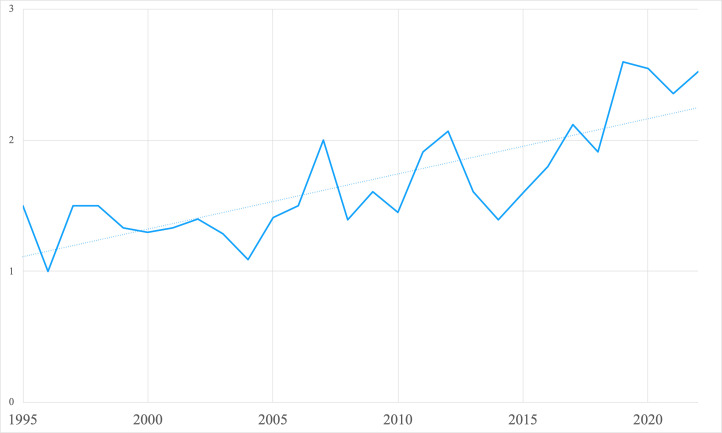
**Panel A.** Collaboration in gender mainstreaming research: average number of authors per article by year. **Panel B.** Annual percentage of articles collaboratively written by North-South authors.

Co-authorship and citation trends presented mixed trends of collaboration and engagement. The steady increase in the number of co-authors per article (**Figure 3**, Panel A), moving beyond the predominance of sole-author publications seen in 1995, suggests greater collaboration among academics, contrasting the grey literature which was often organisation-specific. However, only 6% of the surveyed literature included authors from both the Global North and South. Most articles (75%) were written solely by authors at institutions in the Global North, compared to 19% which were written by authors at Global South institutions only. These collaborations, observed through the percentage of total articles published per year, began to increase modestly around 2009 (**Figure 3**, Panel B).

A discouraging metric is the relatively few times, with some exceptions, that gender mainstreaming articles were cited: 18.4 citations on average per paper (mean of five citations, 15.4% had zero citations) (Figure S1 in the **Online Supplementary Document**).

A limited number of collaborative learning and research programmes – all short-lived – were explicitly discussed in the reviewed literature [5-7]. The Dutch government initiative ‘On Track with Gender,’ for example, conducted surveys and organised a collaborative learning programme that was soon disbanded: ‘The earlier initiative of a Gender Knowledge Platform (*Kenniskring gender en emancipatie*), that was set up to improve knowledge management on gender within the Ministry, is no longer active, primarily because of a lack of time and capacity within the Ministry. The initiative ‘On Track with Gender’ shared a similar fate’ [8].

### Thematic synthesis of gender mainstreaming

#### Key successes

The literature recounts several successes of gender mainstreaming. Principal among these is the widespread endorsement of its use from international nongovernmental organisations, governments, and the development community alongside sustained civil society engagement from feminist and women’s organisations. The literature also describes how the field has moved from theory to practice with the creation and use of new methods and planning frameworks. Case study publications [9] showcase where and how these new frameworks have been effectively adopted.

#### Key challenges

Ultimately, the literature displayed a mixed track record on gender mainstreaming, crystallised in critical reflection on recurring challenges and lament over the field’s unmet potential. Our mapping of the existing research underscored several key challenges (**Table 1**).

**Table 1:** Key challenges in gender mainstreaming identified in literature

Conceptual clarity	Key definitions and concepts are not understood and shared across institutions and practitioners.
**Academic-practitioner disjunctures**	Knowledge sharing and collaboration between practitioners and academics is limited and disincentivised.
**Politics**	Gender mainstreaming can be co-opted or diluted by political actors, straying from its roots in feminist advocacy, and subject to compromises and hollow commitments of resources and political will.
**Leadership and organisational culture**	Efforts are marginalised in terms of funding, staffing, and the organisation’s vision due to active or passive resistance by senior leaders.
**Monitoring and evaluation**	Gender-specific evaluation is often non-mandatory, underfunded, and not fully integrated into existing evaluation frameworks. Lack of measurement hinders the development of a gender mainstreaming evidence base.
**Men’s roles**	An inordinate focus on women’s empowerment can lead to oversight of the roles men must play in policy processes and change initiatives. Different effects of policies and programmes on men can be overlooked.
**Public health sectoral concerns**	Gender mainstreaming has been integrated in an uneven manner into the research and practice of medical specialties and public health areas. Some critics argue that gender mainstreaming is incompatible with health sector objectives and technical capacities due to pragmatic, conceptual, and political reasons. The resulting depoliticisation of gender mainstreaming in public health has led to technocratic solutions or active resistance [10-13].

##### Conceptual clarity

Definitions and objectives of gender mainstreaming vary among institutions, practitioners, and local contexts, resulting in wide-ranging technical approaches and warranting improved clarity [14]. Early conceptualisations of gender and diversity have transitioned from static, binary understandings of gender towards more intersectional approaches [5,15,16]. Despite this theoretical shift, concerns persist about the oversimplification and essentialisation of gender in practice [17,18]. Debate about the ultimate objectives of gender equality, particularly those highlighting the balance of practical vs strategic needs or its framing as an integrationist vs transformative approach, spotlights a perceived divide between gender mainstreaming practice and its roots in feminist theory [5,19-22]. Although flexible interpretations may allow for tailored applications, there is consensus that conceptual ambiguity can impede the development of robust safeguards, effective integration of gender considerations, and empowerment [23-25].

##### Academic-practitioner disjunctures

Higher education and academic disciplines have struggled to incorporate gender analysis into their research and curricula [26-29]. Non-academic knowledge production has also made slow progress: as of 2011, fewer than a quarter of World Health Organization (WHO) publications used sex-disaggregated data [12]. The substantial critical literature closely aligns with the challenges identified by activists and governments [18,30,31]. Despite the agreement on challenges, the gap between academia and practice is perceived as widening, building on historical rifts [32]. Translating feminist theory into practice, ensuring representation in academia, and fostering North-South dialogue engaging both epistemic communities and practice-based networks remain formidable tasks [5,30,32,33]. Some scholars argue that academia also contributes to false success stories in gender equality discourse [18]. Postcolonial feminist theory and transnational thought offer promising avenues for reimagining gender mainstreaming [34].

##### Politics and power

Effective gender mainstreaming typically requires robust governance, including legal recognition of human rights and sufficient state capacity [24]. Gender mainstreaming has consistently faced political struggles, including depoliticisation, underfunding, and superficial inclusion in policy [24,31]. Scholars warn against political dilution and state co-optation of feminist agendas that maintain unequal power relations [35-37]. Unequal power relations have been particularly visible in the vertical (national-local) coordination of gender equality initiatives, in which localisation of policies, engagement with grassroots feminist networks, and co-creation of programmes is often overlooked [38-41]. A body of research addresses ways to remedy these dynamics and demonstrates how the participation of non-state actors is an enabling condition for gender mainstreaming [13,42-47]. Nongovernmental organisations and local actors are disincentivised from addressing political dynamics to maintain access and support from public administration and state institutions, although the extent to which strategic instrumentalism serves rather than harms gender mainstreaming’s aims remains up for debate [7,20,48,49]. Neoliberalism is noted for harnessing gender mainstreaming as a tool to govern gender and uphold the status quo [22], while concurrently shaping a development discourse that limits its ability to effect structural change [18,50].

##### Leadership and organisational culture

Organisational culture, structures, and leadership often undermine gender mainstreaming [7,51,52]. Research underscores the dual need for ‘institutional’ and ‘programmatic’ mainstreaming, with institutional mainstreaming providing an essential foundation for programmatic mainstreaming [53]. Organisational challenges include inadequate financial resources, staff gender imbalances, and non-participatory gender training that set lower expectations for non-gender experts [54,55]. The ‘gender expert’ is heavily scrutinised, with scholars advocating for ‘power and participation’ rather than ‘power and accountability’ [56-58]. Organisations ‘appear to do much,’ but often only undertake surface-level reforms, increasingly delegating gender projects to junior staff or siloed specialised agencies [53,59]. Without broader buy-in, gender initiatives risk being perceived as externally imposed, generating resistance and leading to passive implementation [7]. The bureaucratic handling of gender mainstreaming and prevalence of ‘management by results’ can undermine its transformative potential [60-63]. Scholars call for more research into resistance and institutional learning, coupled with efforts to dismantle the ‘deep structure’ of gender bias in organisations [53,64,65].

##### Monitoring and evaluation

While gender-specific evaluation throughout the policy process is critical to advancing gender mainstreaming, it often remains non-mandatory, underfunded, and not fully integrated into existing evaluation frameworks [66,67]. Lack of measurement and comprehensive gender-disaggregated data hinders the development of a gender mainstreaming evidence base, resulting in best practices that are grounded in practice rather than empirical evidence [53,68,69]. One category of critique, referred to as ‘lost outcomes’ analysis, describes the risk of on-the-ground outcomes being ‘lost’ by overemphasising organisational improvements and dynamics [50,70]. To ensure equitable progress, programme results and gender indicators should be critically examined – for instance, generalised reporting of women’s health gains can obscure disparities across socioeconomic groups [12]. Self-reported performance assessments and minimal repercussions for underperforming institutions remain widespread [53]. However, recent initiatives in international organisations like the 2012 United Nation (UN) System-wide Action Plan (SWAP) on Gender Equality and the Empowerment of Women (GEEW) and new strategies by the WHO and the Pan American Health Organization represent growing accountability [53,55]. Additionally, external monitoring and evaluation organisations (e.g. Global Health 50/50) and tools (e.g. Equilo’s Gender Equality and Social Inclusion Contextual Analysis Tool), law enforcement mechanisms, and donor requirements are growing [24,53,71].

##### Men’s roles

An inordinate focus on women’s empowerment can lead to oversight of the roles men must play in policy processes and change initiatives. Differential impacts of policies and programmes on men are sometimes overlooked despite the shift from ‘Women in Development’ to ‘Gender and Development.’ Preconceived notions about the actors and beneficiaries of gender mainstreaming lead to overlooked allies and inhibit the inclusion of emerging allies and institutions [54]. The 2006 Finnish Presidency of the European Union set evaluating gender equality issues from a male viewpoint as a gender equality priority. Despite this, men within beneficiary communities often feel excluded from and apathetic towards development interventions, interpreting them as ‘women’s empowerment initiatives’ and sometimes prohibiting family members from participating [72]. The policy realm, too, has largely taken gender mainstreaming to refer to women [12]. Scholars note that if gender mainstreaming strives to reduce the gender gap in health outcomes, for example, the recent status of men’s health and observed gaps between male and female life expectancies should cause alarm [12], warranting programming for men. They also contend that gender and development discourse perpetuates gendered assumptions, such as ‘women are less corrupt than men’ or ‘women are more peaceful than men’ [73]. Yet, the broadening of gender mainstreaming participants and sectors risks severing gender mainstreaming from the women’s movement [16].

##### Public health sectoral concerns

During the late 2000s, legislation such as the European Union’s Roadmap for Equality between Women and Men and Health Strategy spurred renewed attention to gender mainstreaming in health [74]. Early emphasis on maternal and reproductive care broadened to address diverse facets of public health as gender-related [11,75-78]. Global health crises such as the human immunodeficiency virus/acquired immunodeficiency syndrome (HIV/AIDS) epidemic were early research focal points [79-81], with recent research spanning neglected tropical diseases, mental health and psychiatry, and Ebola [82-84]. However, academic concerns about fundamental incompatibilities between traditional health contexts and gender mainstreaming amassed as early as 2004. Challenges identified are pragmatic, conceptual, and political, often mirroring challenges faced in gender mainstreaming, health, and policy, and amplified in fragile economies [13,30,85,86]. Critics cite multiple country contexts to assert incongruities between health sector objectives and those of gender mainstreaming and social transformation [12,87,88]. For example, these critics contend that the ‘focus on biological differences’ required to gender mainstream health would ‘emphasise division rather than integration of services’ [16]. They also argue that gender differences in health are uniquely complex compared to other sectors [30]. Gender mainstreaming medical education and health professional training is incomplete, in part due to the gender ratio of the physician workforce [89-91]. Research and data gaps on gender’s impact on care and the social determinants of health contributes to an excessive emphasis on procedures when translating gender mainstreaming into health [16,92].

## DISCUSSION

### Recommendations for an inclusive, collaborative, and structured research agenda

The gender mainstreaming literature has expanded considerably over the last 25 years, yet there remain critical knowledge gaps, theoretical inconsistencies, and implementation challenges. To address them and build upon the existing research, practitioners and scholars must adopt a tactical research agenda. Based on the key challenges identified in the literature, we suggest that taking inclusivity, collaboration, and increased structure as guiding principles for the research process can improve upon past efforts.

#### Inclusive: Elevating voices for equity

Inclusive gender mainstreaming research will require changing ‘who,’ ‘how,’ and ‘where’ research is conducted to co-create knowledge and improve social inclusion. The review highlights leading geographic regions and sectors and the need for greater regional, country, and sector-level diversity in authorship and substantive content. Gender advocates and scholars from the Global South are under-represented in the literature, mirroring north-south disparities present in the broader development literature and current discourse on the need to decolonise global health [93,94].

As others have called for, dedicated research funding and opportunities must be paired with assessment and action on current practices and constraints – geographical, financial, and structural, among others – that exclude and exploit researchers from the Global South [5,93]. Participatory processes that engage local actors and communities in knowledge building can ensure voices at all levels are heard and improve the likelihood of successful policy implementation [95].

Inclusion must be achieved not just in process, but also in a re-envisioning of the gender mainstreaming concept. A full embrace of inclusion requires greater consensus about whether and how gender mainstreaming will incorporate intersectionality. Scholars and practitioners have sought to generate a more expansive and inclusive vision for gender mainstreaming with early efforts ‘focused on the cross-cutting of gender inequalities by ethnicity and class’ and expanding to include other axes of identity such as ‘sexuality, disability, religion, nationality, and age’ [96]. Some have called for a wholesale replacement of gender mainstreaming with an innovative and improved alternative that is intersectional in approach. Questions remain about how gender mainstreaming – in theory, policy, and practice – can fully address these various strands and garner greater attention from the research and practice community.

#### Collaborative: Bridging the research-practice gap

The literature depicts an unsettled role for academic research efforts within the broader gender mainstreaming endeavour, as well as untapped opportunities to bridge the academic-practitioner divide. Though gender mainstreaming grew out of feminist theory, systematic shared learning across practitioners, researchers, and implementing organisations remains limited. This is reflected in the short-lived nature of learning collaboratives for which the primary barrier to their continuation was a lack of dedicated resources, pointing to the need for strategic direction and sustained commitment. As Hankivsky finds in interviews with key stakeholders, ‘it is clear that there is a real disconnection between gender mainstreaming and contemporary theorizing and research’ [97]. The low citation counts identified in the review are also a potential indicator of low readership, engagement, and public impact of gender mainstreaming scholarship.

Significant barriers exist to achieving an effective partnership between academics and practitioners. There are disincentives for non-profit organisations to share internal and proprietary tools, data sets, and learning. Collaboration opportunities are often extractive in nature, demanding practitioners’ time and expertise without sufficient compensation or recognition for those contributions. Improved and recurring feedback loops between the communities can foster practice-relevant research and evidence-based practice. Convening and sustaining these academic-practitioner interactions would require substantial investments of time, funding, and administrative/research capacity.

#### Structured: Making research relevant

Across two and a half decades, the research comprises an echo chamber of gender mainstreaming’s perennial challenges. Simple recognition of these challenges is insufficient to bring about change; future research and practice must push forward and test new solutions and theories of impact, rather than restate existing criticism about gender mainstreaming’s shortcomings. Previous efforts to improve gender mainstreaming have concentrated on pragmatic considerations; a renewed research agenda can articulate and drive action on conceptual and political challenges as well as examine the interrelated nature of the field’s outstanding challenges [30]. Simultaneously, the research must be timely and address new concerns the field faces. Researchers will need to coordinate across ongoing studies to use limited resources strategically, enable greater collaboration, and identify high-level priority research questions.

Besides a structured research agenda, more systematic methodological approaches are needed to build a stronger evidence base. The generalisability of research findings and practitioner use of the findings are undermined by pervasive methodological issues, especially small-scale and non-comparative studies, incomplete data, and narrow evaluation procedures. There is a dearth of systematic comparative analyses, in part because evaluation is underdeveloped [98] and understudied. Measurement and evaluation for relevant gender mainstreaming outcomes generally takes place at the programme or grant level, typically inaccessible to the public or researchers. Existing outcomes data often captures high-level indicators that do not necessarily translate into women’s empowerment more broadly (e.g. increased female representation in politics does not always ensure decreased violence against women). Gender-relevant data are frequently collected in the late stages of project implementation and narrowly focus on final results rather than undertaking continual evaluation which would aid policy design and implementation processes [99]. The evidence base overwhelmingly features small-scale, non-governmental organisation-implemented interventions. Due to the context-specific nature of these interventions, they are less useful for designing government programmes and larger scale initiatives [100]. Furthermore, the vast variations in organisational approaches to gender mainstreaming in use today are more difficult to compare than the limited, common set of frameworks during the early stages of gender mainstreaming [101]. The need for more comparative work is readily acknowledged among scholars [102], with numerous useful comparisons proposed: Between similar countries, between sectors, between different programmes in a single international organisation, or between programmes based on common frameworks or theories of change or different levels of governance and using common evaluation frameworks.

Moreover, sampling techniques are often flawed. Sample sizes are limited, sometimes resulting in researchers drawing conclusions from single cases. The literature is biased towards organisations with relatively established gender mainstreaming policies and programmes, as those organisations are most likely to volunteer to share their experiences or be recruited via snowball sampling techniques. The samples are also often unrepresentative of the contexts where gender mainstreaming occurs. A large body of work addresses the gender mainstreaming experiences of Western countries and institutions, particularly Europe; however, more studies are needed to address ‘how developing countries conceptualise, design and manage gender mainstreaming in development policies and programmes in specific political and economic contexts’ [68].

Encouragingly, scholars are increasingly moving beyond qualitative interviews and document reviews and are incorporating a wider range of methodology and formats (book chapters, articles, and toolkits), including quantitative and policy analysis methods to approach well-established themes in the literature from new angles and build out nascent research areas.

### Building a research pipeline: Work under way and next steps

Existing momentum paired with thoughtful changes to current research practices can achieve a research agenda that is inclusive, collaborative, and structured. Past efforts to improve dialogue across gender mainstreaming stakeholders and research professionals are particularly instructive. The following recommendations build on, rather than replace, long-standing strengths of gender mainstreaming and of academia, such as engaging and empowering regional women’s organisations and working alongside academic institutions and with scholarly journals to develop a research pipeline.

National and regional joint research and advocacy groups could play a larger role in knowledge building and sharing. These groups demonstrate the mobilisation and organisational capacities built by the international women’s movement and could provide important spaces to convene gender experts, facilitate communication, and shape ongoing discourse and policy, given their existing access to local and international development players alike. Their principles of feminist solidarity and commitment to shifting power structures [103] well-position them to advance non-extractive practices and participatory research models. A partnership approach might achieve the dual goals of collective reflection and addressing power dynamics between researchers and gender mainstreaming stakeholders. These groups also have stood the test of time.

Two influential examples are the African Women’s Development and Communications Network (FEMNET) and the Gender and Development Network (GADN) based in the UK. FEMNET is a pan-African feminist network which predates the Beijing conference. Aside from FEMNET’s advocacy work, the >800-member network articulates a clear knowledge production role in its mission statement – ‘to facilitate and coordinate the sharing of experiences, ideas, information, and strategies for human rights promotion among African women’s organizations through networking, communication, capacity-building and advocacy at the regional and international levels’ [104]. They publish the African Women’s Journal, bulletins, and position statements. Founded in 2009, GADN is a newer organisation boasting a membership of over seventy UK-based gender organisations. Both provide models for regionally driven shared learning agendas and working groups. Elevating the profile of regional groups and equipping them with additional skills, resources, and infrastructure to expand their impact, and fold them into research initiatives, should be priorities for the gender and development space.

Another opportunity to build out research pipelines is through gender-attuned editorial policies. Heidari et al. [76] highlight the role of journals and editors in encouraging scholarship that critically engages gender. Editorial guidelines can require or encourage gender analysis or the inclusion of sex-disaggregated data. Individual editors can also adopt a proactive approach. To date, journals may play an awareness-raising role but forego a greater substantive contribution. The outcomes of previous themed journals resulted in the featuring of a select number of academics writing on similar topics and cases to their previous work; this pattern suggests that editorial guidelines and priorities may result in journals going through the motions without provoking or fostering new perspectives on gender mainstreaming. Other academic publishing opportunities are proactive public scholarship to accompany technical journal articles, open access publishing, practitioner article review, and, in more theoretical pieces, greater attention to practical implications and generalisability.

Finally, practitioners and academics should continue to advocate for gender mainstreaming topics to be taught in higher education programmes, such as political science and public affairs curricula and health professional training. Gender mainstreaming gained recognition as an important policy priority among international organisations yet is still a marginalised lens in mainstream international relations and academia. In fact, scholars who prioritise gender research risk professional setbacks, creating a vicious cycle. Describing the process of mainstreaming political science, Atchinson [105] states:

While there are no systematic data on the employment of gender and politics scholars, it is not rare for a gender and politics scholar to be told that her/his employment prospects would be better if she/he were to research something else (Childs and Krook, 2006). Given the position of women in the profession and the status of gender and politics in the discipline, it is unsurprising that there is continued resistance to integrating gender into mainstream political science education.

Gender mainstreaming should not be disciplinarily siloed. Gender is deeply relevant to broad-ranging fields such as environmental science, economics, and urban design, among others, and scholars from these and other fields would benefit from training on how to integrate gender mainstreaming principles into their work. Such training would help ensure that sufficient data are being collected for gender experts to analyse.

### Strengths and limitations

A strength of this review is the extensive literature, diversity of sectors, and dates of publication it encompasses. Yet there are also several limitations. The Scopus citation counts that we analysed are derived only from journals covered by Scopus; however, this platform offers more expansive journal coverage than some other sources for citation analysis, such as Web of Science, and omits citations from types of publications (e.g. dissertations/theses) that may be less relevant to our analysis. It also may have excluded technical reports or policy reports of potential interest. The review’s exclusion of foreign language literature may overlook the research productivity of countries where English is not the primary academic publishing language. Moreover, ‘Gender’ and ‘mainstreaming,’ are broad terms intended to capture learning in the field and are unlikely to identify sectoral case studies with titles excluding ‘mainstreaming.’ Case studies, reports, and working papers are also published by regional and country headquarters for development organisations and are uncaptured in these trends. The selection process for full review incorporated several criteria but was nonetheless subjective and may not have captured all relevant articles. Our approach to assessing Global North-South collaboration neglects some nuance, such as diasporic scholars or Global North institutions within the Global South, and simplifies development into binary categories; nevertheless, the dearth of collaboration between North and South institutions was apparent.

## CONCLUSIONS

Gender mainstreaming has made considerable strides, but development organisations – and advocates for women’s advancement more broadly – face formidable and new challenges, including recovery from the coronavirus disease 2019 (COVID-19) pandemic and ongoing global racial injustice. Historically, shocks to the global system – such as the 2008 recession or major changes to the foreign aid landscape – considerably impacted gender equality policies and programming, often for the worse. The rapidly changing global context makes an up-to-date literature review and reflection on the status of gender mainstreaming all the more urgent. Can gender and development practitioners meet the moment?

This scoping and thematic review highlights that the increased scholarly attention towards gender mainstreaming has yet to result in a cohesive evidence base moving the field forward. However, increased awareness of gender and social inclusion has yielded greater data, toolkits, and frameworks for action at international and domestic levels. Despite its fragmented nature, existing scholarship showcases accomplishment, often in small-scale interventions. These accomplishments inspire cautious optimism that gender mainstreaming in both scholarship and practice can mature and actualise its greater objective of achieving gender equality. An inclusive, collaborative, and structured research agenda can better leverage academia to assist practitioners and supporters in realising gender mainstreaming’s relevance and potential for impact across health and development sectors.

## Additional material


Online Supplementary Document
