# Supplementary material for: Gender mainstreaming at 25 years: Toward an inclusive, collaborative, and structured research agenda
Source: J Glob Health. 2024 Jan 26;14:04011. doi: 10.7189/jogh.14.04011 (PMC10811564; doi:10.7189/jogh.14.04011)

Supplemental Materials

Gender mainstreaming at 25 years: toward an inclusive, collaborative, and structured research agenda

Kelsi Caywood, Gary L. Darmstadt

Table of Contents

**Table S1.** Top funding sources for gender mainstreaming publications	1
**Table S2.** University affiliations of authors of gender mainstreaming articles	2
**Table S3.** Geographic context or institutional actor of focus of gender mainstreaming articles	3
**Table S4.** Evolving trends in sectors of focus in gender mainstreaming literature over time	4
**Figure S1.** Distribution of gender mainstreaming articles by citation ranges	5

Table S1. Top funding sources for gender mainstreaming publications

Funding Source	Number of Articles
UK Economic and Social Research Council	10
European Commission	6
Horizon 2020 Framework Programme	5
Social Sciences and Humanities Research Council of Canada	5
Academy of Finland	3
Consortium of International Agricultural Research Centers	3
UK Department for International Development	3
World Bank Group	3

Table S2. University affiliations of authors of gender mainstreaming articles

University	Number of Articles
Radboud University	15
Simon Fraser University	9
Cardiff University	8
University of the West of England	7
University of Liverpool	7
Complutense University of Madrid	7
London School of Economics and Political Science	6
University of Auckland	6
University of Antwerp	6
Royal Tropical Institute - KIT	6

Note: tabulations include co-author affiliations.

Table S3. Geographic context or institutional actor of focus of gender mainstreaming articles

Focus	Number of Articles
European Union and Europe-wide	82
United Kingdom	33
United Nations system	32
Sweden	23
India	20
Indonesia	19
Germany	16
Uganda	14
Canada	13
South Africa	13

Note: articles without a specific geographic region are excluded from the tabulation.

Table S4. Evolving trends in sectors of focus in gender mainstreaming literature over time

Focus	Article Count
**1995-2009**	
Law and Policy	18
Health	17
Labor, employment, and the workplace	11
Security, national militaries, peacekeeping	10
Governance and politics	10
WASH	9
Education	7
Natural disasters and management	5
Architecture, design, and urban planning	5
Organizations	4
**2010-2022**	
Governance and politics	46
Health	33
Education	31
Security, national militaries, peacekeeping	26
Climate change and environment	20
Law and policy	17
Architecture, design, and urban planning	16
Agriculture	15
Natural disasters and management	12
WASH	12

Figure S1. Distribution of gender mainstreaming articles by citation ranges

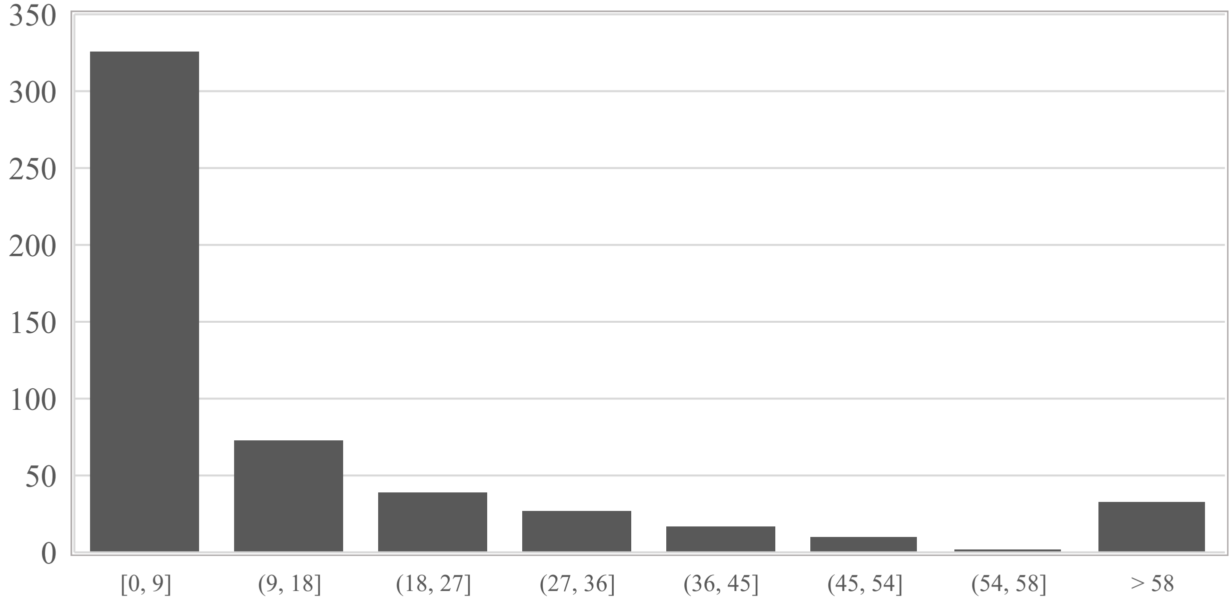

Supplement: Online Supplementary Document [file jogh-14-04011-s001.pdf]
